# 

**Published:** 1880-04

**Authors:** 


					﻿whole Number,
CCXXV.
APRIL, 1880.
Number 9,
Volume XIX.
THE
BUFFALO
Medical and Surgical Journal.
EDITORS:
THOS. I.OTHROP, M. I).,
A. R. DAVIDSON, M. D.,
I». W. VAN PF.Y.1IA, M. !>.,
HERMAN MVNTER, M. I>.,
I.l’CIEN HOWE, M. I).
BUFFALO :
Published by the Medical Journal Association.
Read the Notice of this Journal among the Advertisements.
Entered at the Post-Office at Buffalo, N. Y., as Second-Class Mail Matter.
				

